# Incidence of Emergency Department Visits for Sexual Abuse Among Youth in Hong Kong Before and During the COVID-19 Pandemic

**DOI:** 10.1001/jamanetworkopen.2022.36278

**Published:** 2022-10-20

**Authors:** Janet Yuen Ha Wong, Luke Y. F. Luk, Tsz Fung Yip, Teddy Tai Loy Lee, Abraham Ka Chung Wai, Joshua W. K. Ho

**Affiliations:** 1 School of Nursing and Health Studies, Hong Kong Metropolitan University, Hong Kong SAR, China; 2 School of Nursing, Li Ka Shing Faculty of Medicine, The University of Hong Kong, Pokfulam, Hong Kong SAR, China; 3 School of Biomedical Sciences, Li Ka Shing Faculty of Medicine, The University of Hong Kong, Pokfulam, Hong Kong SAR, China; 4 Laboratory of Data Discovery for Health Limited (D^2^4H), Hong Kong Science Park, Hong Kong SAR, China; 5 Department of Emergency Medicine, School of Clinical Medicine, Li Ka Shing Faculty of Medicine, The University of Hong Kong, Pokfulam, Hong Kong SAR, China; 6 Department of Accident and Emergency, The University of Hong Kong–Shenzhen Hospital, Shenzhen, China

## Abstract

This cohort study assesses the incidence of emergency department (ED) visits in Hong Kong, China, for sexual abuse among youth before and during the COVID-19 pandemic.

## Introduction

Extended school closures and restricted social activities during the COVID-19 pandemic may have increased the likelihood of youth being exposed to child sexual abuse (CSA), as seen during previous public health emergencies.^1^ Nonetheless, quantitative data are lacking. The aim of the study was to compare the incidence of CSA in youth before and during the COVID-19 pandemic in Hong Kong.

## Methods

This cohort study analyzed 1 493 833 records of children and adolescents (age <18 years) from 18 emergency departments (EDs) in Hong Kong, China, between 2016 and 2021 that were extracted from Hong Kong Hospital Authority’s administrative database. The University of Hong Kong and Hospital Authority Hong Kong West Cluster institutional review board approved the study and waived the informed consent requirement because only deidentified data were used. We followed the STROBE reporting guideline.

Sexual abuse was identified based on *International Classification of Diseases, Ninth Revision, Clinical Modification* code 995.53. Because CSA reporting is often followed by an ED referral in Hong Kong, the data provide a representative sample of all reported CSA cases. Hong Kong’s first confirmed COVID-19 case was in January 2020, and social distancing measures, including approximately 10 months of complete or partial school closure, were implemented. Emergency department visits for CSA between January 1, 2020, and December 31, 2021 (pandemic period), were compared with those between January 1, 2016, and December 31, 2019 (prepandemic period). We developed a negative binomial model of monthly incidence rate of CSA (count per 1000 youth^2^; eTable in the Supplement) that accounts for the pandemic period, patient sex, and their interaction. Relative incidence ratios (RIRs) across time periods were used as estimates of relative changes in CSA cases (eMethods in the Supplement). The threshold for statistical significance was *P* < .05. We observed patterns among key subgroups, including sex, age, and period of school closure and reopening. Statistical analysis was performed using R software, version 4.1.0 (R Foundation for Statistical Computing).

## Results

**Figure 1.**
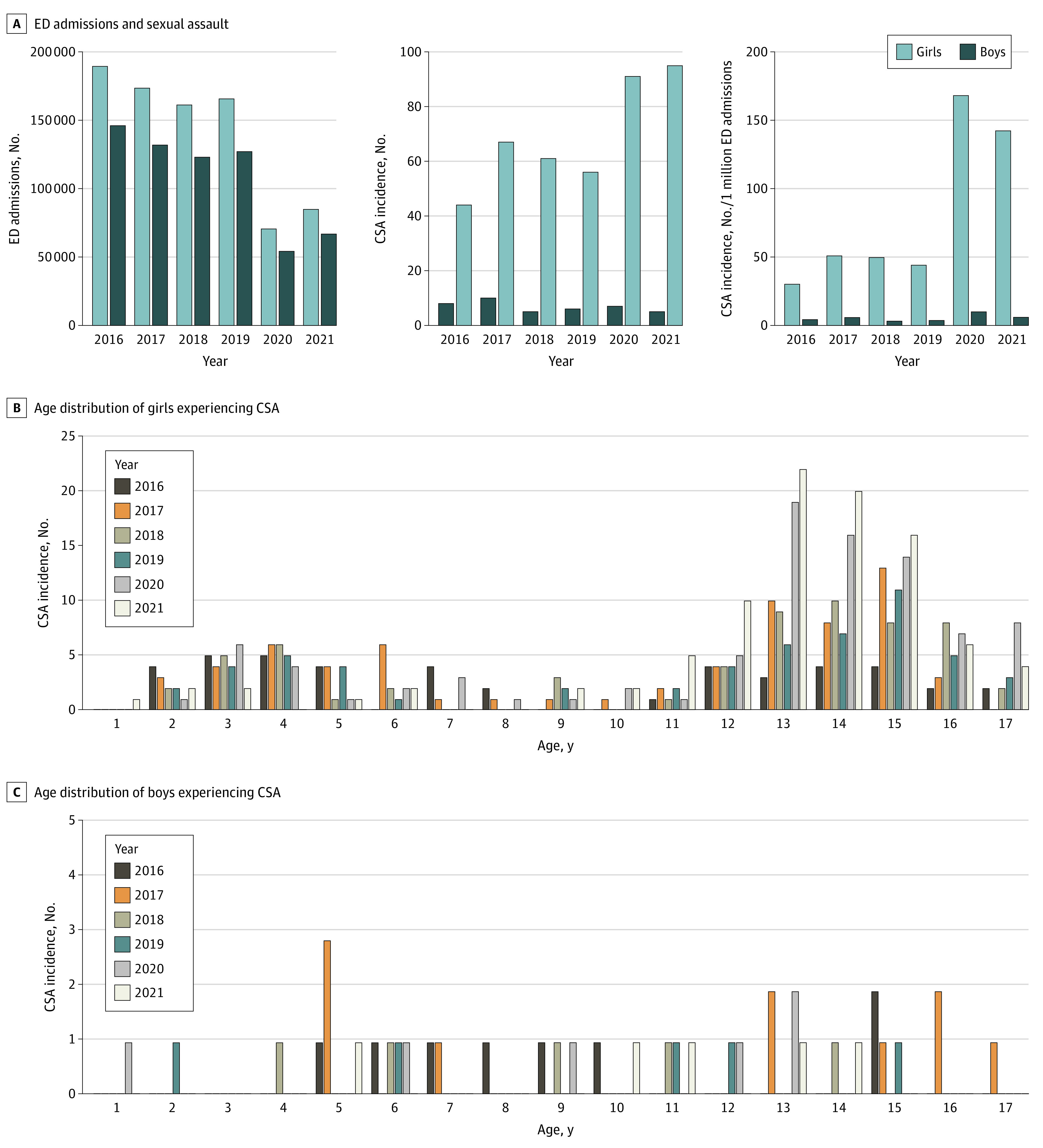
Sex and Age Distribution of Children Who Presented to Hong Kong Emergency Departments Between 2016 and 2021 CSA indicates child sexual abuse; ED, emergency department.

**Figure 2.**
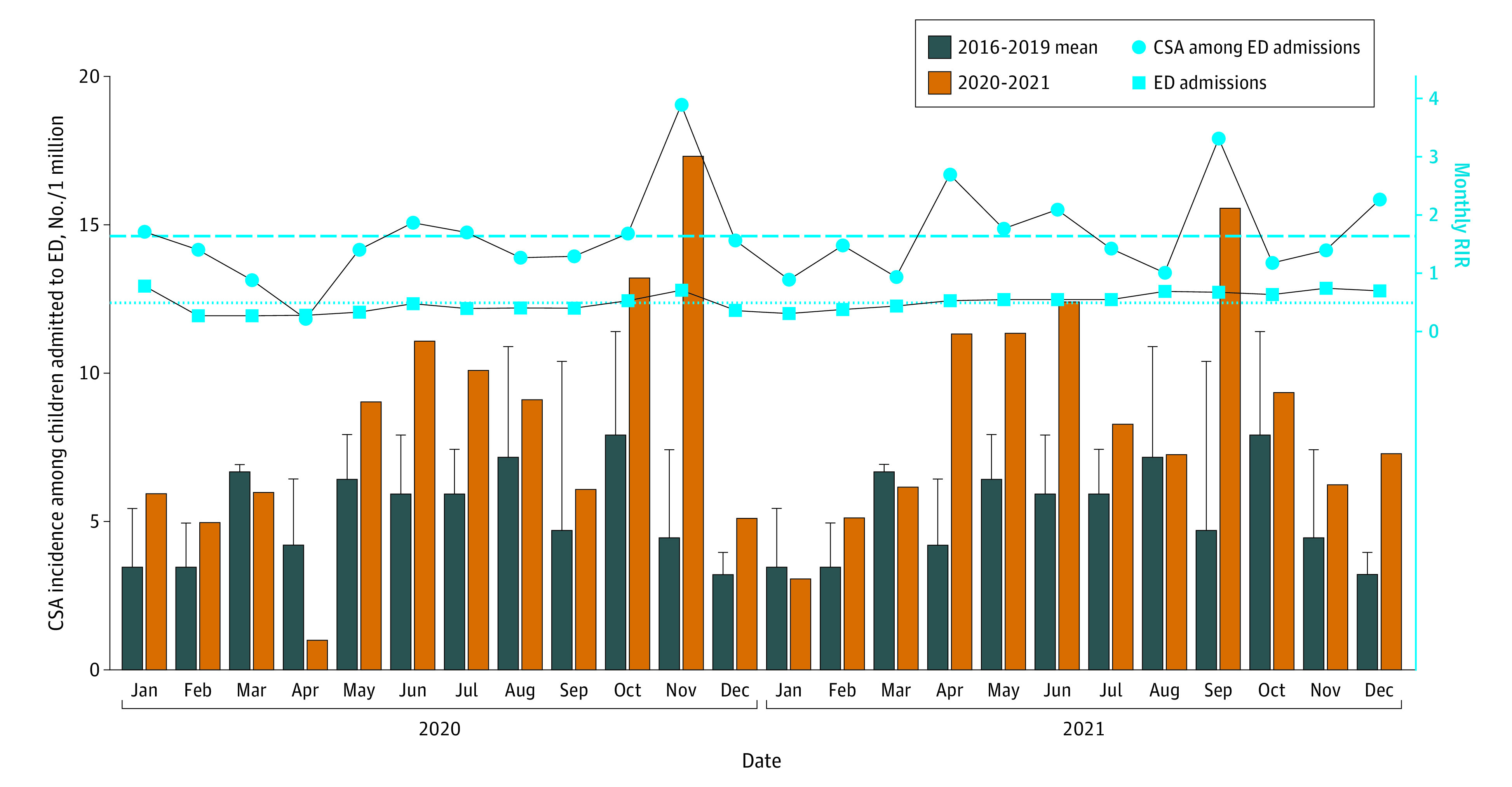
Monthly Incidence Rate of Child Sexual Abuse During COVID-19 Pandemic Period (2020-2021) vs Prepandemic Period (2016-2019) in Hong Kong CSA indicates child sexual abuse; ED, emergency department.

Annual youth ED visits decreased 54.6% after the start of the pandemic (prepandemic mean [SD], 304 390 [10 640.77]; pandemic mean [SD], 138 137 [19 059.36]) (Figure 1A). Child sexual abuse was identified in 455 encounters of 427 patients: 414 cases involving 387 girls (90.6%) vs 41 cases involving 40 boys (9.4%) (Figure 1A). Median patient age was 13 years (median absolute deviation, 2.97). Incidence rate per million girls significantly increased (monthly mean, 9.7 prepandemic cases vs 16.28 pandemic cases; RIR, 1.68; 95% CI, 1.32-2.13; *P* < .001), mostly among girls aged 12 to 17 years (Figure 1B). There was no significant change in CSA cases in boys per million (monthly mean, 1.16 prepandemic cases vs 0.99 pandemic cases; RIR, 0.86; 95% CI, 0.43-1.70; *P* = .66; Figure 1C). Although the number of CSA cases increased throughout the 24-month pandemic period, an increase often occurred immediately after resumption of face-to-face classes in secondary schools (Figure 2).

## Discussion

Despite the overall reduction in ED visits among youth in Hong Kong,^3^ findings of this large cohort study suggest an increase in reported CSA cases during the pandemic vs prepandemic periods. The data suggest that teenage girls were more likely to experience sexual abuse during the pandemic; this observation is consistent with local official statistics.^4^ Because most CSA was committed by a family member of the patient,^5^ extended school closures may have limited CSA detection and reporting, which may explain the observed surge in CSA cases immediately after school resumption. Therefore, we support prosecuting bystanders who fail to take reasonable steps to protect youth from CSA and introducing an online reporting system.^6^ A limitation of this study was that data captured only patients who presented to an ED after experiencing CSA. Also, our results may not be generalizable to low- and middle-income countries. Our analysis suggests the possibility that prolonged public health interventions for infection containment may be followed by other serious health and social outcomes.^1,5^

## Notes

Supplement. **eTable.** Hong Kong’s Population Aged 0-17 Years From 2016 to 2021 **eMethods.** Details of the Statistical Model
