# Supplementary material for: Incidence of Emergency Department Visits for Sexual Abuse Among Youth in Hong Kong Before and During the COVID-19 Pandemic
Source: JAMA Netw Open. 2022 Oct 20;5(10):e2236278. doi: 10.1001/jamanetworkopen.2022.36278 (PMC9585429; doi:10.1001/jamanetworkopen.2022.36278)
Supplement: Supplement. — eTable. Hong Kong’s Population Aged 0-17 Years From 2016 to 2021 eMethods. Details of the Statistical Model [file jamanetwopen-e2236278-s001.pdf]

Supplementary Online Content

Wong JYH, Luk LYF, Yip TF, et al. Incidence of emergency department visits for sexual abuse among youth in Hong Kong before and during the COVID-19 pandemic. *JAMA Netw Open.* 2022;5(10):e2236278. doi:10.1001/jamanetworkopen.2022.36278

eTable. Hong Kong's Population Aged 0-17 Years From 2016 to 2021
eMethods. Details of the Statistical Model

This supplementary material has been provided by the authors to give readers additional information about their work.

© 2022 Wong JYH et al. *JAMA Network Open.*

eTable 1. Hong Kong's Population Aged 0-17 Years From 2016 to 2021

		Population aged 0-17 (in thousands)		
**Year**	**Reference time-point**	**Male**	**Female**	**Both sexes**
**2015**	Year-end	522.3	488.7	1,011
**2016**	Mid-year	524.6	491.2	1,015.8
**2016**	Year-end	524	489.8	1,013.8
**2017**	Mid-year	523	489.1	1,012.1
**2017**	Year-end	520.3	487.5	1,007.8
**2018**	Mid-year	522.4	490.5	1,012.9
**2018**	Year-end	519.4	488.4	1,007.8
**2019**	Mid-year	520	489.8	1,009.8
**2019**	Year-end	521.7	492.6	1,014.3
**2020**	Mid-year	509.9	483.1	993
**2020**	Year-end	503.4	476.7	980.1
**2021**	Mid-year	497.7	469.9	967.6
**2021**	Year-end p	494.6	466.1	960.7

p: Provisional figure

The data in this table was obtained from the website of the Census and Statistics Department of the Government of Hong Kong Special Administrative Region. Between 2016 and 2021, there were approximately 500,000 male and 500,000 female population aged between 0-17. In the analysis, we have chosen 500,000 (for sex-specific analysis) and 1 million (for all sex) as the base for presenting incidence rates. Monthly counts were estimated based on linear interpolation of yearly and mid-yearly counts.

© 2022 Wong JYH et al. *JAMA Network Open.*

eMethods. Details of the Statistical Model

In Hong Kong, of the availability of public primary care services do not meet the demand, so there is a disproportional number of people, especially elderly people, who use Emergency Departments (ED) of public hospitals for non-emergent medical concerns. This means the total ED visits contains true medical emergency and non-emergent cases that should have been better served via primary heart services. During the COVID-19 periods (2020-2021), the vast majority of reduction of ED visits are from patients with a non-emergent and semi-urgent triage category.(1) There is almost no reduction in the critical and urgent cases. Child sexual assault (CSA) cases are usually referred to the ED by the police for medical examination as part of their routine investigation. Therefore, we believe using population size as a denominator is much more reasonable than total ED visits.

Nonetheless, for completeness, we have performed the analysis to show the estimated RIR of CSA cases with (1) total paediatric population, and (2) total ED visits. The conclusions were similar when using ED visits as the denominator, but due to the limitation of word count, we have limited the results in the paper to those using the population denominator.

The exposure in our model is a dummy variable (the pandemic term) representing COVID 19 status. We have included sex as a covariate and included its interaction with time period in the model. The response variable Y in our negative binomial model is monthly counts and population is the offset (the “denominator”). This model does not include age and month. The monthly count for males and some age groups (pre-teen) can be quite small (see Figure 1b and 1c for annual count by sex and age), so the RIR estimates will likely have high variability. Nonetheless, for completeness of the analysis, we developed a larger model which includes age, interactions with age, time trends and months. We then tested all possible sub-models based on the following combinations of choice of terms:

1. Degree of the ‘age’ polynomial terms, which range between 1 and 7, and their interactions with pandemic term and sex term.
2. Inclusion of the ‘month’ term
3. Inclusion of the ‘trend’ term

This gives 7*2*2=28 sub-models. Furthermore, we repeated this analysis using (1) total paediatric population or (2) total ED visits as the off-set (see response to the previous question). As a result, we fitted 28*2=56 sub-models.

This is the expected result for our data distribution, as the effect of age on CSA cases appears to be nonlinear and varies with sex (Figure 1b) and the linear term for age alone does not fit the data well.

1. Wai AKC, Wong CKH, Wong JYH, Xiong X, Chu OCK, Wong MS, et al. Changes in Emergency Department Visits, Diagnostic Groups, and 28-Day Mortality Associated With the COVID-19 Pandemic: A Territory-Wide, Retrospective, Cohort Study. Annals of Emergency Medicine. 2022;79(2):148-57.

© 2022 Wong JYH et al. *JAMA Network Open.*
